# LncRNA ANRIL regulates AML development through modulating the glucose metabolism pathway of AdipoR1/AMPK/SIRT1

**DOI:** 10.1186/s12943-018-0879-9

**Published:** 2018-08-22

**Authors:** Lin-Yu Sun, Xiao-Juan Li, Yu-Meng Sun, Wei Huang, Ke Fang, Cai Han, Zhen-Hua Chen, Xue-Qun Luo, Yue-Qin Chen, Wen-Tao Wang

**Affiliations:** 1 Key Laboratory of Gene Engineering of the Ministry of Education, State Key Laboratory for Biocontrol, Sun Yat-sen University, Guangzhou, 510275 China; 2 The First Affiliated Hospital of Sun Yat-sen University, Guangzhou, 510080 China; 3 School of Life Science, Sun Yat-sen University, Guangzhou, 510275 People’s Republic of China

**Keywords:** AML, ANRIL, Glucose metabolism, AdipoR1

## Abstract

The long noncoding RNA ANRIL has been found to be abnormally expressed and play important roles in different cancers. However, the expression and function of ANRIL in acute myeloid leukemia (AML) remain to be declared. In this study, we found that ANRIL is up-regulated in AML patients at diagnosis and down-regulated in patients after complete remission (CR). Functional studies showed that knockdown of ANRIL expression resulted in a decline in glucose uptake and inhibition of AML cell maintenance in vitro and in vivo. Mechanically, ANRIL was found to repress the expression of Adiponectin receptor (AdipoR1), a key regulator of glucose metabolism. Both ANRIL and AdipoR1 knockdown reduced the expression levels of phosphorylation of AMPK and SIRT1, implying a previously unappreciated ANRIL-AdipoR1-AMPK/SIRT1 signaling pathway in regulating cell glucose metabolism and survival in AML. The study is the first to demonstrate that ANRIL promotes malignant cell survival and cell glucose metabolism to accelerate AML progression and is a potential prognostic marker and therapeutic target in AML treatment.

**Electronic supplementary material:**

The online version of this article (10.1186/s12943-018-0879-9) contains supplementary material, which is available to authorized users.

AML is a heterogeneous malignancy characterized by uncontrollable proliferation of leukemia cells in bone marrow, and accounts for 30% of leukemia-related pediatric deaths [1, 2]. Despite recent progress in treating AML, its long-term survival is still poor due to the development of resistance and the high rates of relapse after treatment with the currently available chemotherapy [1, 2]. Thus, finding novel therapy targets is urgently needed to improve the clinical outcomes of AML.

In recent years, long non-coding RNAs (lncRNAs) have been found to be dysregulated in cancer [3–5]. However, lncRNAs that regulate AML development and progression remain largely unstudied. In our previous study, we noted that an lncRNA, ANRIL (antisense non-coding RNA at the *INK4* locus), was highly expressed in acute leukemia patients compared to that in normal controls [6]. ANRIL was reported to repress the expression of p15^INK4B^ and p16^INK4A^ in multiple solid tumors [3, 7]. More interestingly, ANRIL was recently found to transcriptionally suppress AdipoR1 in atherosclerosis and periodontitis [7]. AdipoR1 is a key protein that is intimately involved with cell senescence and metabolism [7, 8]. Therefore, we hypothesized that ANRIL is involved in AML progression via regulating cell metabolism pathways.

## Results and discussion

### ANRIL is significantly higher expressed in patient samples and regulates cell survival in AML

**Fig. 1:**
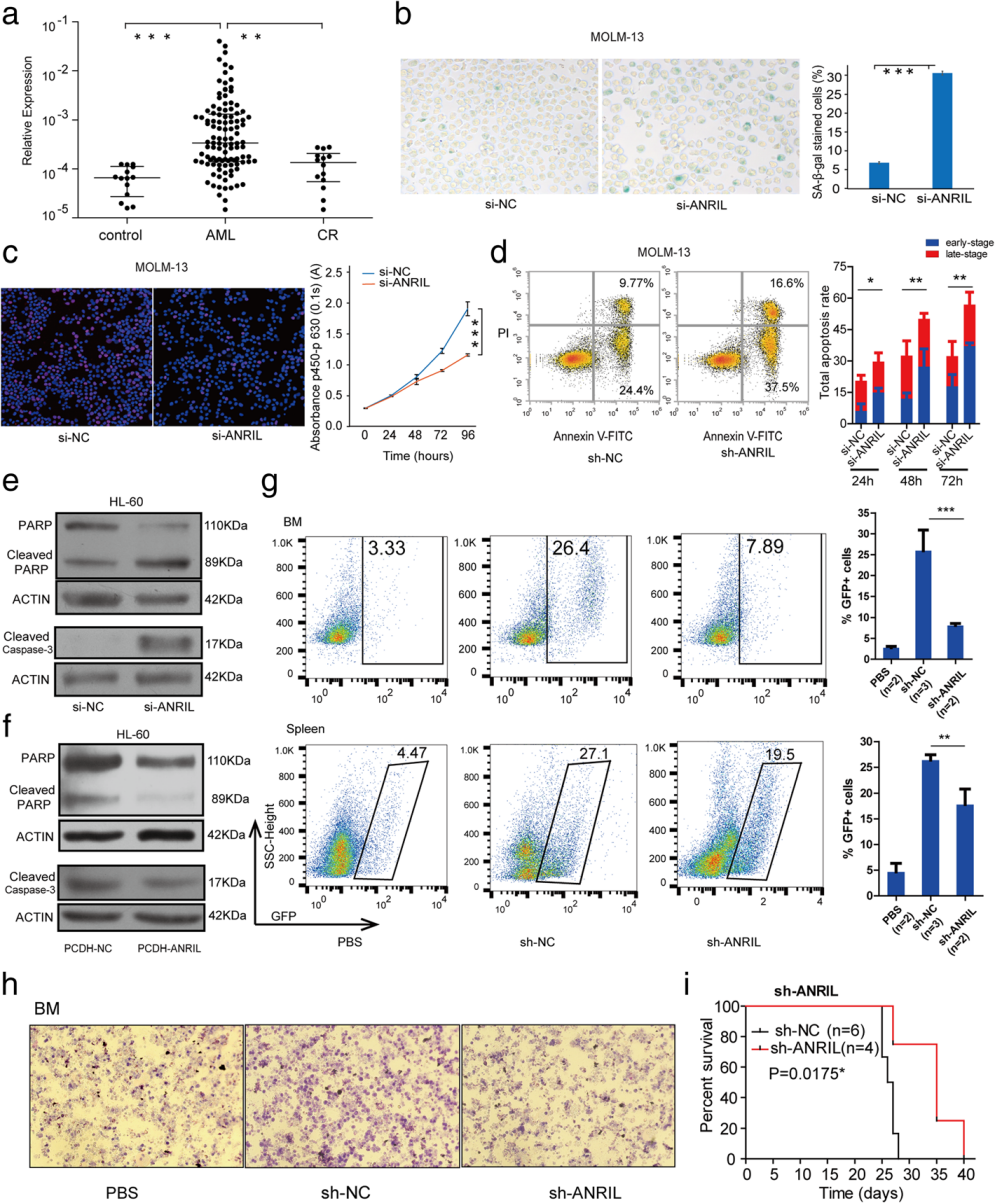
ANRIL is significantly highly expressed in AML patient samples and regulates AML progression in vitro and in vivo. **a** The expression of ANRIL in AML patients was detected by qRT-PCR, **p < 0.01,***p < 0.001. **b** Knockdown of ANRIL can induce cell senescence in MOLM-13 cells, ***p < 0.001. **c** The cell proliferation detected using CCK-8 and Edu assays, respectively, in MOLM-13 lines were blocked when ANRIL knocked down, ***p < 0.001. **d** ANRIL knocked down enhanced ATO-induced cell apoptosis in a time course (24 h, 48 h, 72 h) in MOLM-13 cells, *p < 0.05, ***p < 0.01. The representative photograph of flow cytometry was shown. **e** The western blot for the cleaved PARP and caspase 3 upon knockdown of ANRIL in HL60 cells. **f** The western blot for the expression levels of cleaved PARP and caspase 3 under the overexpression of ANRIL. **g** NOD-*scid*-mice model that intravenously (tail vein) implanted by sh-NC and sh-ANRIL established MOLM-13 cells, the percentages of GFP+ MOLM-13 cells were checked in BM, and spleen after 3 weeks implantation. Error bars reflect ±SEM (*, *p* < 0.05). **h** Bone marrow smear showed the amount of GFP+ MOLM-13 cells from sh-ANRIL group after transplantation. **i** Kaplan–Meier curves showed the survival of the sh-NC and sh-ANRIL established mice (*, *p* < 0.05)

To validate the expression pattern of ANRIL in AML, we recruited 109 with newly diagnosed AML and 14 controls to evaluate the clinical relevance of ANRIL. The detailed clinical parameters are presented in Additional file 1: Table S1. As shown in Fig. 1a, the expression level of ANRIL was remarkably increased in AML patients in different stages of AML compared with normal controls, and significantly decreased in CR patients (Fig. 1a and Additional file 1: Figure S1a), suggesting that ANRIL might function as an oncogene in AML.

To demonstrate the oncogenic role of ANRIL, we applied RNA interference (RNAi) to knock down the expression of ANRIL in AML cell lines HL-60 and MOLM-13 (Additional file 1: Figure S1b) and investigated the effects of ANRIL on cell functions. As shown in Fig. 1b and Additional file 1: Figure S1c, senescence cells stained with SA-β-gal were increased after ANRIL knockdown, indicating that down-regulation of ANRIL was able to promote cell senescence. Similarly, the repression of ANRIL resulted in a remarkable decrease in AML cell growth compared with the negative control (Fig. 1c and Additional file 1: Figure S1d). In addition, we also detected the impact of ANRIL on apoptosis induced by ATO (arsenic trioxide), a chemotherapeutic drug in AML treatment, and found that knockdown of ANRIL facilitated ATO-induced cell apoptosis in a time course (24 h, 48 h, 72 h) in AML cells (Fig. 1d and Additional file 1: Figure S1e). We also detected two major apoptosis-related factors, caspase-3 and PARP, and found the cleaved PARP and caspase 3 were significantly increased/decreased upon knockdown/overexpression of ANRIL, respectively (Fig. 1e, f and Additional file 1: Figure S1f, g). Together, these data indicated that ANRIL indeed functions as an oncogene in AML and could regulate leukemic cell survival.

### ANRIL promotes AML progression in vivo

Next, a mouse model was used to further validate the function of ANRIL in vivo*.* MOLM-13 cells transfected with ANRIL short hairpin RNA (named as sh-ANRIL) and negative control short hairpin RNA (named as sh-NC) were intravenously (tail vein) implanted into NOD/SCID mice. The knockdown efficiency was shown in Additional file 1: Figure S2a. We killed the mice after 3 weeks and found that the percentages of GFP+ MOLM-13 cells in BM, peripheral blood and spleen were significantly lower in the ANRIL-knockdown mice than that in sh-NC mice (Fig. 1g and Additional file 1: Figure S2b). Furthermore, bone marrow smear results also showed that the amount of GFP+ MOLM-13 cells from sh-ANRIL group decreased after transplantation (Fig. 1h). Notably, the sh-ANRIL groups survived longer than the control groups (Fig. 1i), suggesting that ANRIL-knockdown could inhibit AML maintenance.

### ANRIL affects a number of genes involved in AML cell metabolism pathways

**Fig. 2:**
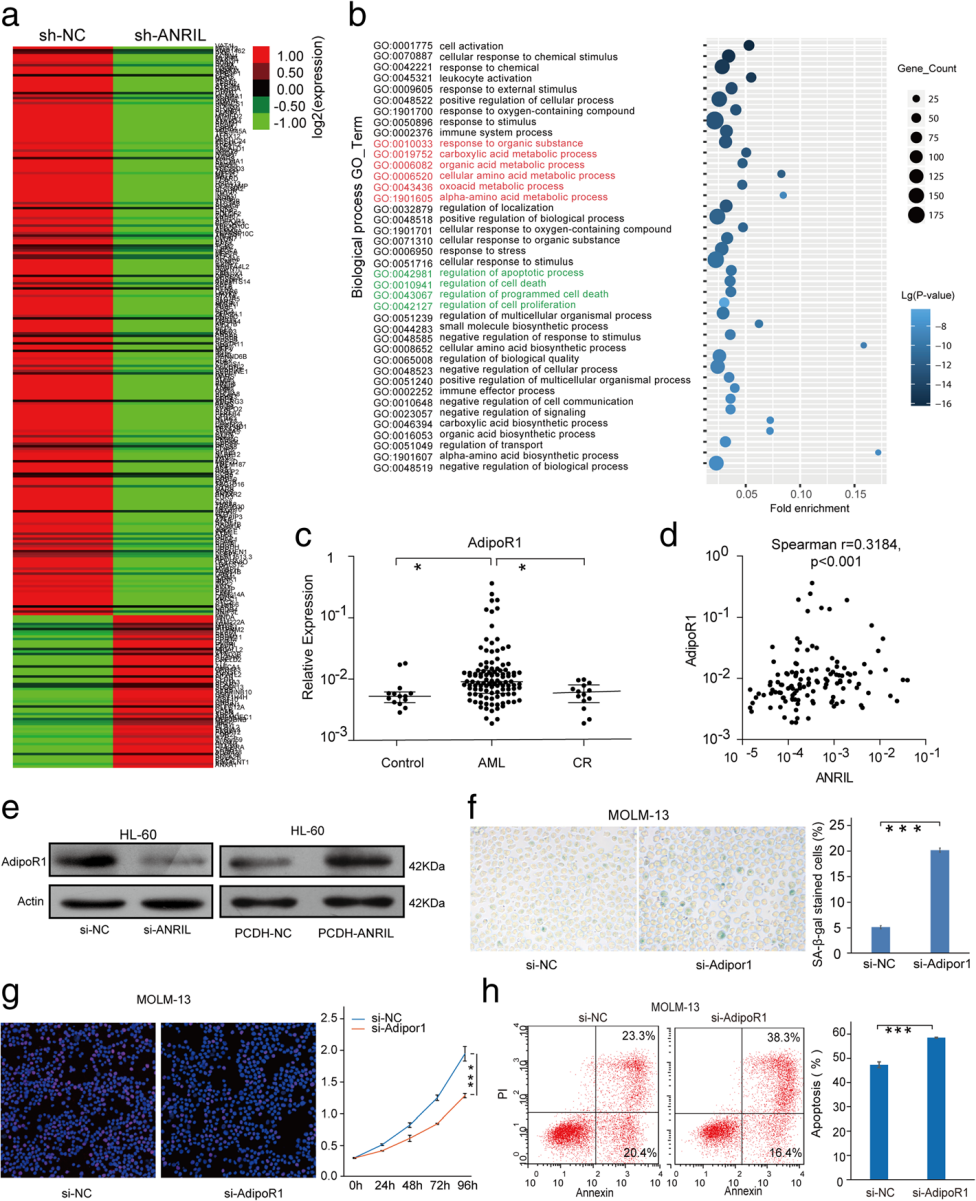
ANRIL regulates cell senescence and apoptosis by regulating the expression of AdipoR1 in AML. **a** Heat maps showed the most differentially expressed mRNAs (fold-change> 2.0) between sh-ANRIL and sh-NC samples. **b** GO analysis annotates the biological process and clusters the modules of genes.The top 40 cluster ranked by *p* value was shown. **c** qRT-PCR results for the expression of AdipoR1 in AML patients at diagnosis and remission, as well as in control samples, *p < 0.05. **d** Spearman correlation analysis indicated the considerably positive relationship between ANRIL and AdipoR1 expression in AML patients (Spearman *r* = 0.3184, *p* < 0.001). **e** The expression of AdipoR1 upon knockdown or overexpression of ANRIL in HL60 cells. **f** Knockdown of AdipoR1 can induce cell senescence in AML cells, ***p < 0.001. **g** Si-AdipoR1 blocked cell proliferation detected using CCK-8 and Edu assays in AML cells, ***p < 0.001. **h** Downregulated AdipoR1 enhanced ATO-induced cell apoptosis in AML cells, ***p < 0.001

We then investigated the underlying mechanism for ANRIL-mediated regulation of AML progression. Using the RNA-seq approaches, we analyzed and compared the expression pattern of mRNAs from the MOLM-13 cells tranfected with sh-ANRIL and sh-NC, and found a set of mRNAs expressed abnormally in sh-ANRIL MOLM-13 cells compared with sh-NC cells (Additional file 1: Figure S3). The Fig. 2a showed the most differentially expressed ones (fold-change> 2.0) between sh-ANRIL and sh-NC, which include 60 mRNAs significantly up-regulated and 228 mRNAs significantly down-regulated in sh-ANRIL MOLM-13 cells. We used GO analysis to annotate biological process and clustered the modules of genes. Notably, as shown in the top 40 cluster ranked by *p* value, the “Metabolic process” (Marked in red) and “cell death regulation” (Marked in blue) were significantly enriched (Fig. 2b), suggesting that the lncRNA ANRIL might regulate the AML cell survival through modulating the leukemic cell metabolism.

### ANRIL regulates AdipoR1 to trigger cell survival in AML

Among the down-regulated genes affected by ANRIL shown in Fig. 2a, AdipoR1 attracted our interesting. AdipoR1 was found to be transcriptionally suppressed by ANRIL in atherosclerosis, periodontitis [7]. Thus, we asked if ANRIL regulated AML cell survival by targeting AdipoR1. We first detected the expression of AdipoR1 in AML patient samples, and found that the expression of AdipoR1 was significantly up-regulated in AML patients compared with that in normal controls, and down-regulated in patients who have achieved CR (Fig. 2c). The expression of AdipoR1 was positively correlated with ANRIL levels (Fig. 2d).The knockdown or overexpression of ANRIL resulted in the down- or up-regulation of AdipoR1 protein level (Fig. 2e and Additional file 1: Figure S4a), suggesting an association between ANRIL and AdipoR1 in AML. Silence of AdipoR1 expression could promote cell senescence, apoptosis, and decreased cell proliferation, which are similar to the effects of ANRIL (Fig. 2f-h and Additional file 1: Figure S4b-g). These data together demonstrated that ANRIL regulates the expression of AdipoR1 in AML.

### ANRIL regulates cell survival through a glucose metabolism pathway of AdipoR1/AMPK/SIRT1

**Fig. 3:**
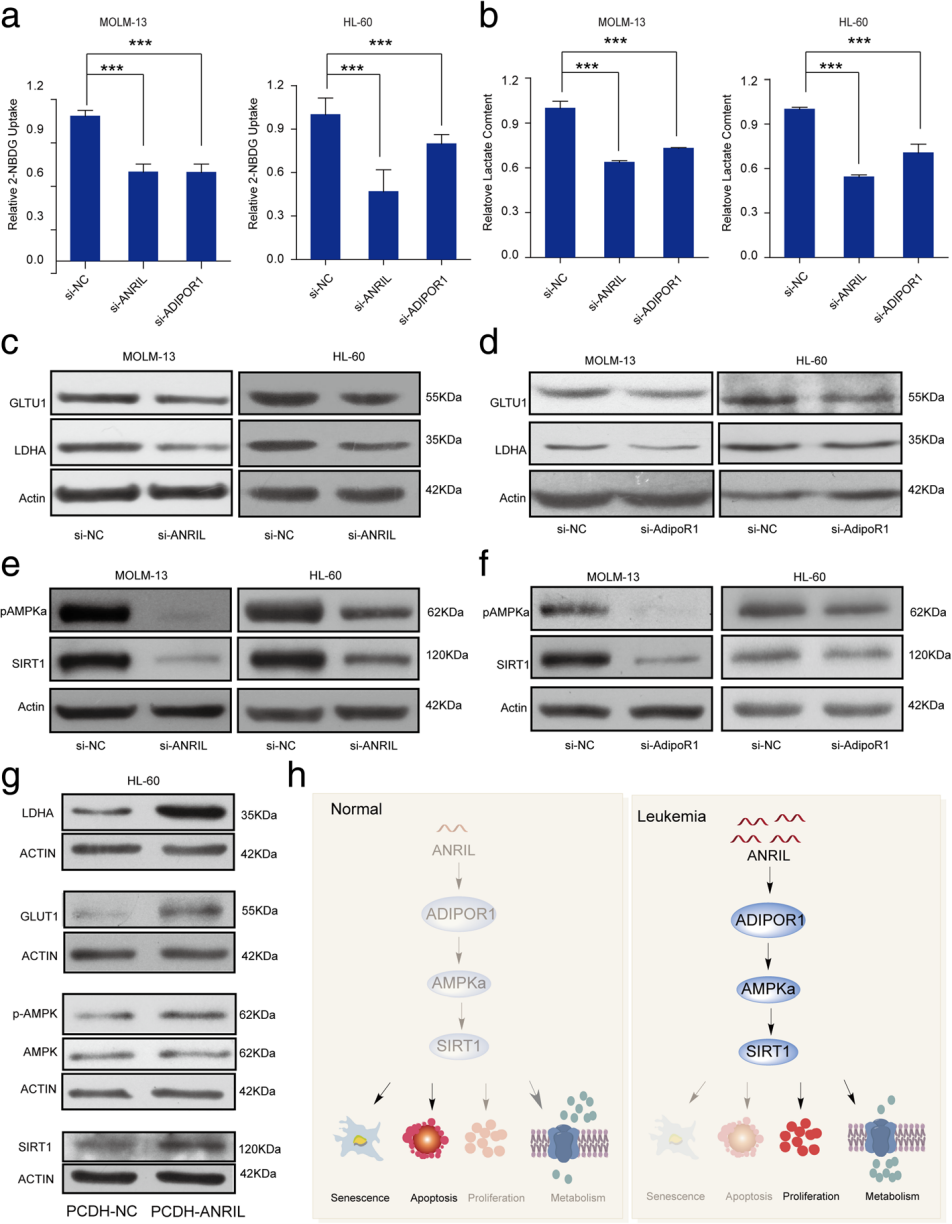
ANRIL regulates glucose metabolism through the AdiopR1/AMPK/SIRT1 signaling pathway. **a** The glucose uptake after the transfection of si-ANRIL and si-AdipoR1 in MOLM-13 and HL-60 cells. **b** The relative levels of lactate after the transfection of si-ANRIL and si-AdipoR1 in MOLM-13 and HL-60 cells. The protein expression of GLUT1 and LDHA were decreased after the transfection of siRNA against ANRIL (**c**) and AdipoR1 (**d**) respectively in AML cells. The protein expression of AMPK, p-AMPK and SIRT1 were significantly decreased after the transfection of siRNA against ANRIL (**e**) and AdipoR1 (**f**) respectively in AML cells. **g** Western blot results shown the protein levels of LDHA, GLUT1, SIRT1, the total AMPK and pAMPK when we overexpressed ANRIL in HL60 cells. **h** The proposed working model: in normal cells, ANRIL has a lower expression level;While in AML patients, ANRIL was found to be aberrantly highly expressed and acts as an oncogene via regulating cell senescence and glucose metabolism

AdipoR1 has been demonstrated as a regulator in cell metabolism, such as Warburg effect that plays a pivotal role in cancer [7–9]. Therefore, we further investigated whether ANRIL enhances AML progression by regulating cell metabolism. As shown in Fig. 3a, knocking down both ANRIL and AdiopR1 resulted in a decline in glucose uptake in AML cells. Additionally, the levels of lactate in the culture medium declined after transfection of siRNA against ANRIL or AdipoR1 in AML cells (Fig. 3b), showing that both ANRIL and AdipoR1 are involved in the glucose metabolism of AML. To further confirm the regulation of ANRIL and AdipoR1 in glucose metabolism, we subsequently detected the expression of glucose transporter 1 (GLUT1), a glucose transporter that mediates the transportation of glucose across the plasma membrane of cells, and lactate dehydrogenase A (LDHA), a key enzyme that catalyzes the last step of glycolysis to convert pyruvate to lactate [9]. These results showed that the expression of GLUT1 and LDHA decreased significantly upon knockdown of ANRIL (Fig. 3c) and AdipoR1 in AML cells (Fig. 3d). We next explored the cell glucose metabolism pathways that ANRIL might be involved in. AMPK and SIRT1, regulated by AdipoR1, are crucial targets in AML treatment and the main regulators of cell senescence and cell metabolism [8–10]. The expression of pAMPK (Thr172), the activated form of AMPK, and SIRT1 were concurrently decreased in AML cells once ANRIL and AdipoR1 were knocked down (Fig. 3e, f and Additional file 1: Figure S5a, b). Furthermore, the expression levels of pAMPK, SIRT1, GLUT1 and LDHA were increased when forced expression of ANRIL in AML cells (Fig. 3g). Finally, immunohistochemistry assay showed decreases of the ADIPOR1, pAMPKa and SIRT1 protein levels in the BM of the sh-ANRIL-Molm13 mice compared to those in the sh-NC-Molm13 control mice (Additional file 1:Figure S5c). These results illustrated that ANRIL could function as an oncogene in AML and regulate cell survival through a glucose metabolism signaling pathways of AdipoR1/AMPK/SIRT1 as shown in Fig. 3h.

## Conclusions

ANRIL was found to be aberrantly expressed in AML patients and regulates the disease development through modulating the glucose metabolism pathway. As summarized in Fig. 3h, the function of ANRIL is realized by regulating a key regulator of glucose metabolism named AdipoR1 and its downstream factors AMPK/SIRT1 in AML. The study suggests that the specific expression pattern of ANRIL could serve as a promising target for AML diagnosis and treatment.

## Additional file

Additional file 1: **Figure S1.** ANRIL regulates AML progression in vitro. **Figure S2**. ANRIL affects AML progression *in vivo*. **Figure S3.** Volcano plot based on differential mRNA profiles between sh-NC and sh-ANRIL established MOLM-13 cells. **Figure S4.** ANRIL regulates AML progression in vitro. **Figure S5**. ANRIL regulates the AdiopR1/AMPK/SIRT1 signaling pathway. **Table S1.** Characteristics of test cohort. (DOCX 4562 kb)

## Abbreviations

AdipoR1: Adiponectin receptor 1
ALL: Acute lymphoblastic leukemia
AML: Acute myeloid leukemia
ANRIL: Antisense non-coding RNA in the INK4 locus
CR: Complete remission
GLUT1: Glucose transporter 1
LDHA: Lactate dehydrogenase A
lncRNA: Long noncoding RNA
qRT-PCR: Quantitative real-time PCR
RNAi: RNA interference
