# Supplementary material for: LncRNA ANRIL regulates AML development through modulating the glucose metabolism pathway of AdipoR1/AMPK/SIRT1
Source: Mol Cancer. 2018 Aug 22;17:127. doi: 10.1186/s12943-018-0879-9 (PMC6106744; doi:10.1186/s12943-018-0879-9)
Supplement: Supplementary file 1 — Figure S1. ANRIL regulates AML progression in vitro. Figure S2. ANRIL affects AML progression in vivo. Figure S3. Volcano plot based on differential mRNA profiles between sh-NC and sh-ANRIL established MOLM-13 cells. Figure S4. ANRIL regulates AML progression in vitro. Figure S5. ANRIL regulates the AdiopR1/AMPK/SIRT1 signaling pathway. Table S1. Characteristics of test cohort. (DOCX 4562 kb) [file 12943_2018_879_MOESM1_ESM.docx]

**Additional files**

**Material Method**

**Patients and samples**

A total of 123 bone marrow samples of AML patients were used, including 109 AML samples at primary diagnosis and 14 AML samples at complete remission, as well as 14 bone marrow samples of ITP patients, which served as the control cohort. The detailed clinical parameters are presented in **Table S1.** All the samples were from the First Affiliated Hospital of Sun Yat-sen University. This study was approved by the ethics committee of the affiliated hospitals of Sun Yat-sen University.

**Cell lines and cultures**

MOLM-13 and HL-60 leukemia cells were purchased from American Type Culture Collection (ATCC, Manassas, VA, USA) and were cultured in modified RPMI Medium (HyClone, South Logan, UT, USA). All media contained 10% fetal bovine serum (Gibco, ThermoFisher Scientific, Waltham, MA, USA). The cells were cultured in a humidified atmosphere containing 5% CO2 at 37 °C.

**RNA isolation and quantitative real-time PCR**

Total RNA was isolated from the bone marrow cells using TRIzol reagent (Invitrogen, Carlsbad, USA) and reversed transcribed to cDNA using a PrimeScript RT reagent kit with gDNA Eraser (Perfect Real Time) (Takara, Tokyo, Japan). The real-time PCR was performed using SYBR Premix Ex Taq (TliRNaseH Plus) (Takara, Tokyo, Japan) according to the manufacturer’s instructions. ANRIL expression was detected using the following primers: ANRIL (5’-AGAAAGGAAAGCGAGGTCATC-3’, 5’-GCGTGCAGCGGTTTAGTTTA-3’); AdipoR1 5’-CTTCTACTGCTCCCCACAGC -3’, 5’-GACAAAGCCCTCAGCGATAG-3’; and Actin (5’-TTGTTACAGGAAGTCCCTTGCC-3’, 5’-ATGCTATCACCTCCCCTGTGTG-3’.

**Transient transfection**

Small interfering RNAs (siRNAs) against human ANRIL and AdipoR1 transcripts and a negative control RNA duplex (denoted as control siRNA) were purchased from Guangzhou RiboBio Co., Ltd. Recombinant vectors encoding human ANRIL (NR_120536.1) were constructed by PCR-based amplification (ANRIL-F-FULL: 5’-AGCTACATCCGTCACCTGACA-3’, ANRIL-R-FULL: 5’-TTTATTGTCTGAGCCCAGTTTTATTTTGT-3’) from the cDNA of human HL60 cells and then subcloned into the pCDH-CMV-MCS-EF1-Puro-copGFP eukaryotic expression vector (Addgene, USA). MOLM-13 and HL-60 cells were transfected with siRNAs using the Neon Transfection System (Invitrogen, Carlsbad, USA) according to the manufacturer’s guidelines.

**Western blotting**

Proteins were extracted using RIPA buffer (Beyotime, Shanghai, China), separated on an SDS–polyacrylamide electrophoresis gel and then transferred to a polyvinylidene fluoride membrane (Millipore, MA, USA). The antibodies used in this study are anti-AMPKa (Cell Signalling Technology, Boston, USA), anti-pAMPKa (Cell Signalling Technology, Boston, USA), anti-PARP (Cell Signalling Technology, Boston, USA), anti-Cleaved Caspase-3(Asp175) (Cell Signalling Technology, Boston, USA), anti-AdipoR1 (Abcam, Cambridge, UK), anti-SIRT1 (Santa Cruz, Paso Robles, CA, USA), and anti-β-actin (Sigma-Aldrich, St. Louis, USA) antibodies.

**Proliferation assays**

Cell proliferation was assessed using the Cell Counting Kit-8 (CCK-8) (Dojindo Molecular Technologies, Shanghai, China). A total of 1*10^4^ cells per well were incubated in 96-well culture plates after transfection. The culture medium was changed after 0, 24, 48, 72 and 96 hours.

In addition, a Cell-Light™ EdU Apollo®567 In Vitro Imaging Kit (Guangzhou Ribo-Bio, Guangzhou, China) was used to detect cell proliferation. Transfected cells were incubated with 50 µM EdU for 1 hour and washed twice with PBS. Cells were harvested and fixed with formaldehyde at room temperature for 30 minutes and then neutralized with formaldehyde by glycine for 5 minutes. Cells were stained by incubating Apollo staining mix at room temperature for 30 minutes and permeated by 0.5% Triton X-100 for 10 minutes. Stained cells can be observed with a fluorescence microscope.

**Apoptosis assays**

For the apoptosis assay, cells after transfection were harvested and washed with PBS. The Annexin V-PI Kit (Nanjing Keygen, Nanjing, China) was used according to the manufacturer's guidelines and analyzed by a FACS Calibur using CellQuest software (BD, San Jose, CA, USA).

**β-galactosidase senescence assays**

A β-galactosidase senescence assay was performed with a Senescence β-Galactosidase Staining Kit (Beyotime, Shanghai, China). Cells were harvested after 72 h of transfection, washed with PBS, and then fixed with 4% paraformaldehyde at room temperature for 15 min. After incubation with senescence-associated β-galactosidase staining solution overnight at 37°C, senescent cells were counted under a light microscope.

**Glucose uptake assay**

A glucose uptake assay was performed with a Glucose Uptake Cell-Based Assay Kit (Cayman chemical, USA). Cells were cultured in RPMI-1640 culture medium without glucose for 4 hours after transfection for 60 hours. Next, the cells were washed twice with a cell-based assay buffer after incubation with 150 ng/ml 2-NBDG for 10 min at 37°C. Cells were diluted at the same concentration and analyzed using a fluorescence microplate.

**Lactate Production Assay**

Transfected cells were cultured for 72 h, and the lactate level in the culture medium was analyzed using a lactate assay kit (Cayman chemical, USA) according to the manufacturer’s instructions

**Animal model**

Five-week-old male NOD-SCID mice were maintained under specific pathogen-free conditions in the Laboratory Animal Center of Sun Yat-sen University. All experimental procedures were performed according to the institutional ethical guidelines for animal experiments. Mice were randomly assigned to two groups. In each group, lentiviral stably transduced sh-NC, sh-ANRIL cells were intravenously (tail vein) implanted. Direct injection of 5×106 shRNA transformed MOLM-13 cells in 150 μL of PBS was performed to establish intravenous (tail vein) leukemia. The xenografted mice were randomized into different group. For the control, 150 μL of PBS without cells was injected. We killed the mice in each group after 3 weeks. Subsequently, the peripheral blood, spleen and BM from xenograft mice were treated with a red blood cell lysis buffer (Biolegend, USA). Flow cytometry for the GFP+ % of transduced MOLM-13 cells was performed on a C6 cytometer (BD, USA) and analyzed using FlowJo software. The mice left were performed the survival assay.

**Statistical analysis**

All statistical calculations were performed using SPSS PASW Statistics (version 17.0) and figures were imaged using GraphPad Prism (version 5.0). For clinic data analysis, Mann-Whitney U test were used to determine the significance of differentially expressed lncRNA and mRNA levels between two groups. The Kruskal-Wallis test was used when the comparison was made among 3 groups of lncRNA and mRNA from leukemia patients, and multiple comparisons were made with a LSD-t test. The P value less than 0.05 were considered significant. For cellular experimental data analysis, all the experiments were performed in triplicate, and all of the results were measured as the mean ± SD. The differences between the two groups were analyzed by a 2-tailed Student’s t-test and a difference of P <0.05 was considered significant. For Gene Ontology (GO) analysis, we used the biological processes and molecular function categories in the Gene Ontology Consortium database ([http://www.geneontology.org](http://www.geneontology.org/)).


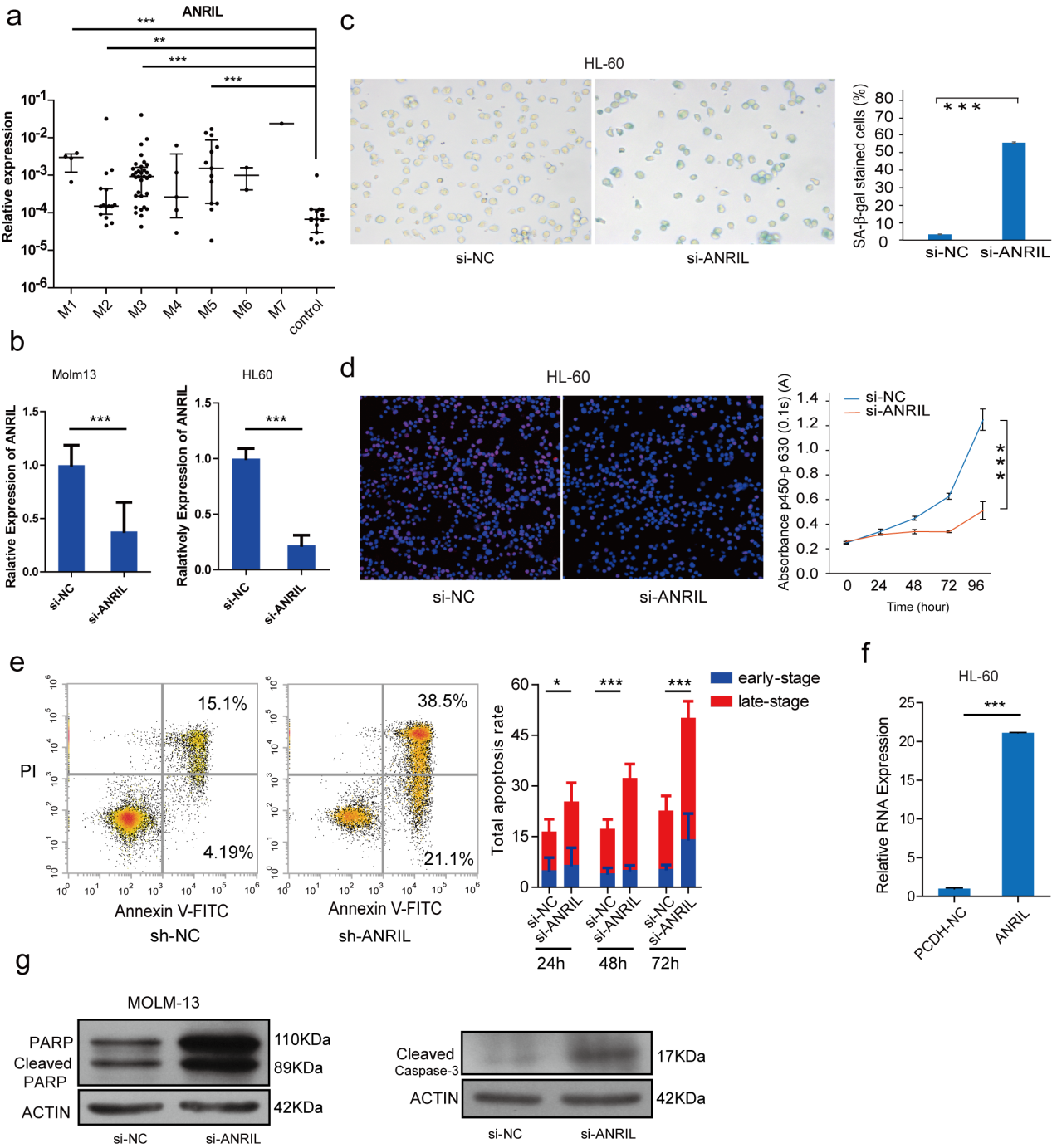


**Figure S1 ANRIL regulates AML progression in vitro.**

**a** The expression levels of ANRIL in different stages of AML (FAB M1-M7). The Kruskal-Wallis test was used to compare all groups of lncRNA from leukemia patients, and multiple comparisons were made with a LSD-t test. **p<0.01, ***p<0.001. **b** The efficiency of siRNA against ANRIL in AML cells. **c** Knockdown of ANRIL can induce cell senescence in MOLM-13 cells, ***p<0.001. **d** The cell proliferation detected using CCK-8 and Edu assays, respectively, in MOLM-13 lines were blocked when ANRIL knocked down, ***p<0.001. **e** ANRIL knocked down enhanced ATO-induced cell apoptosis in a time course (24h, 48h, 72h) in HL-60 cells, *p<0.05, **p<0.01. The representative photograph of flow cytometry was shown. **f** The qPCR for the relative expression of ANRIL by transfecting ANRIL-overexpressed plasmid in HL60 cells, ***p<0.001. **g** The western blot for the cleaved PARP and caspase 3 upon knockdown of ANRIL in Molm13 cells.

**
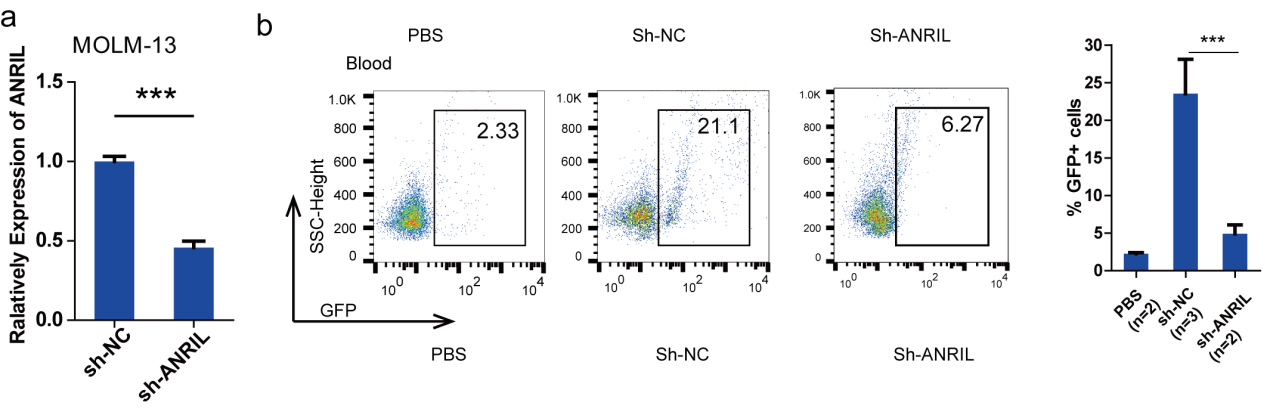
**

**Figure S2 The** **ANRIL affects AML progression *in vivo*.** **a** The efficiency of shRNA against ANRIL in MOLM-13 cells. **b** The percentages of GFP+ MOLM-13 cells in blood.

**
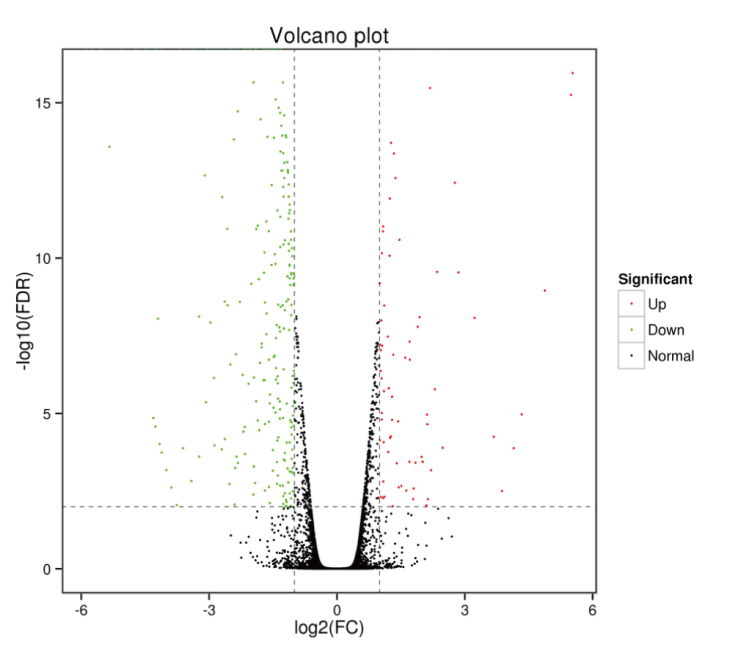
**

**Figure S3 Volcano plot based on differential mRNA profiles between sh-NC and sh-ANRIL** **established MOLM-13 cells.**

**
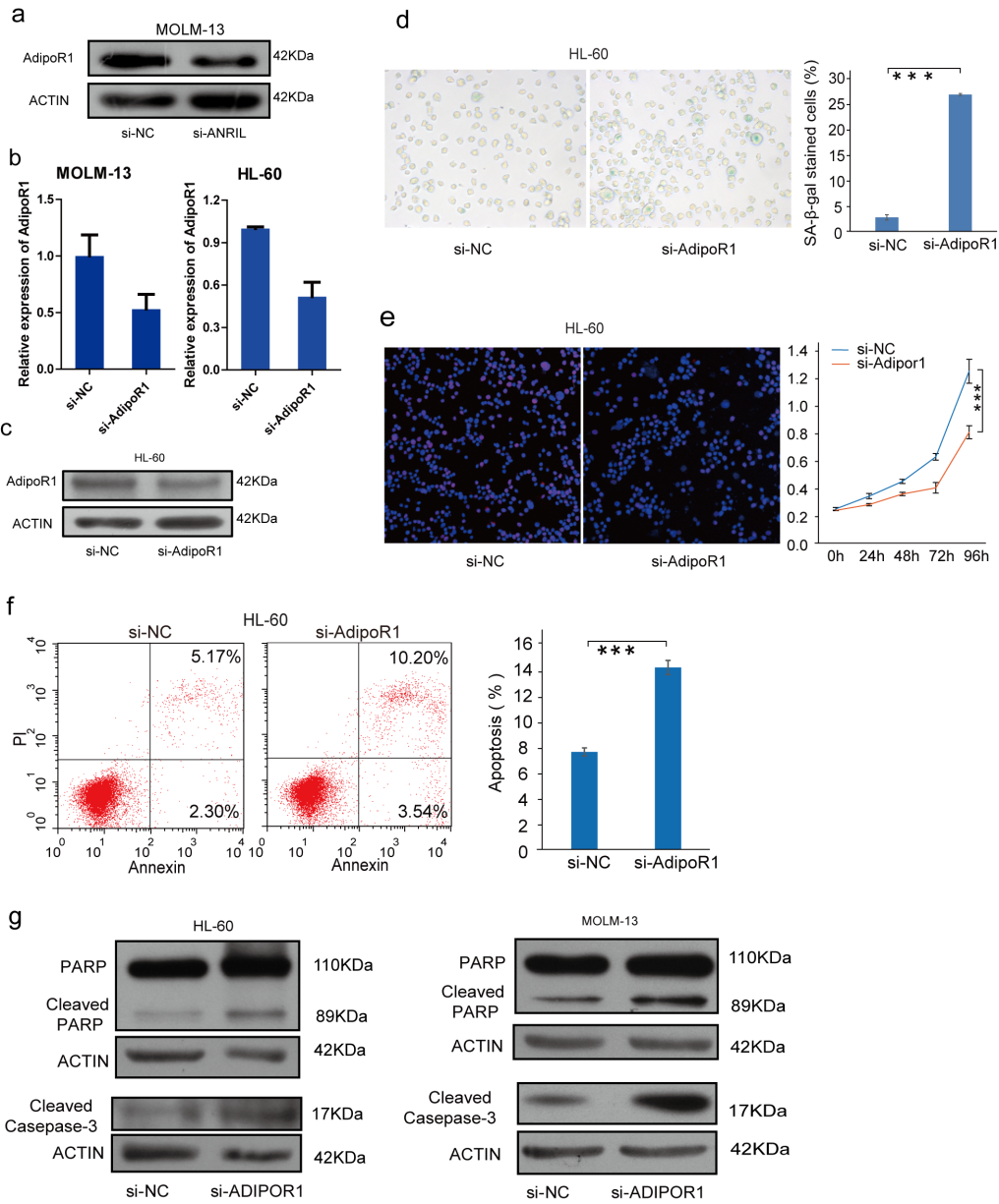
**

**Figure S4 ANRIL regulates promotes AML progression in vitro.** **a** The western blot for the AdipoR1 under ANRIL knocking down. **b** qRT-PCR results have shown the efficiency of siRNA against AdipoR1. **c** The efficiency of siRNA against AdipoR1 detected by western blot assay. **d** Knockdown of AdipoR1 can induce cell senescence in HL60 cells, ***p<0.001. **e** Si-AdipoR1 blocked cell proliferation in HL60 cells, ***p<0.001, and cell proliferation was detected using CCK-8 and Edu assays. **f** Downregulated AdipoR1 enhanced ATO-induced cell apoptosis in HL60 cells, ***p<0.001. **g** The western blot for the expression levels of cleaved PARP and caspase 3 under the knockdown of ANRIL in AML cells.

**
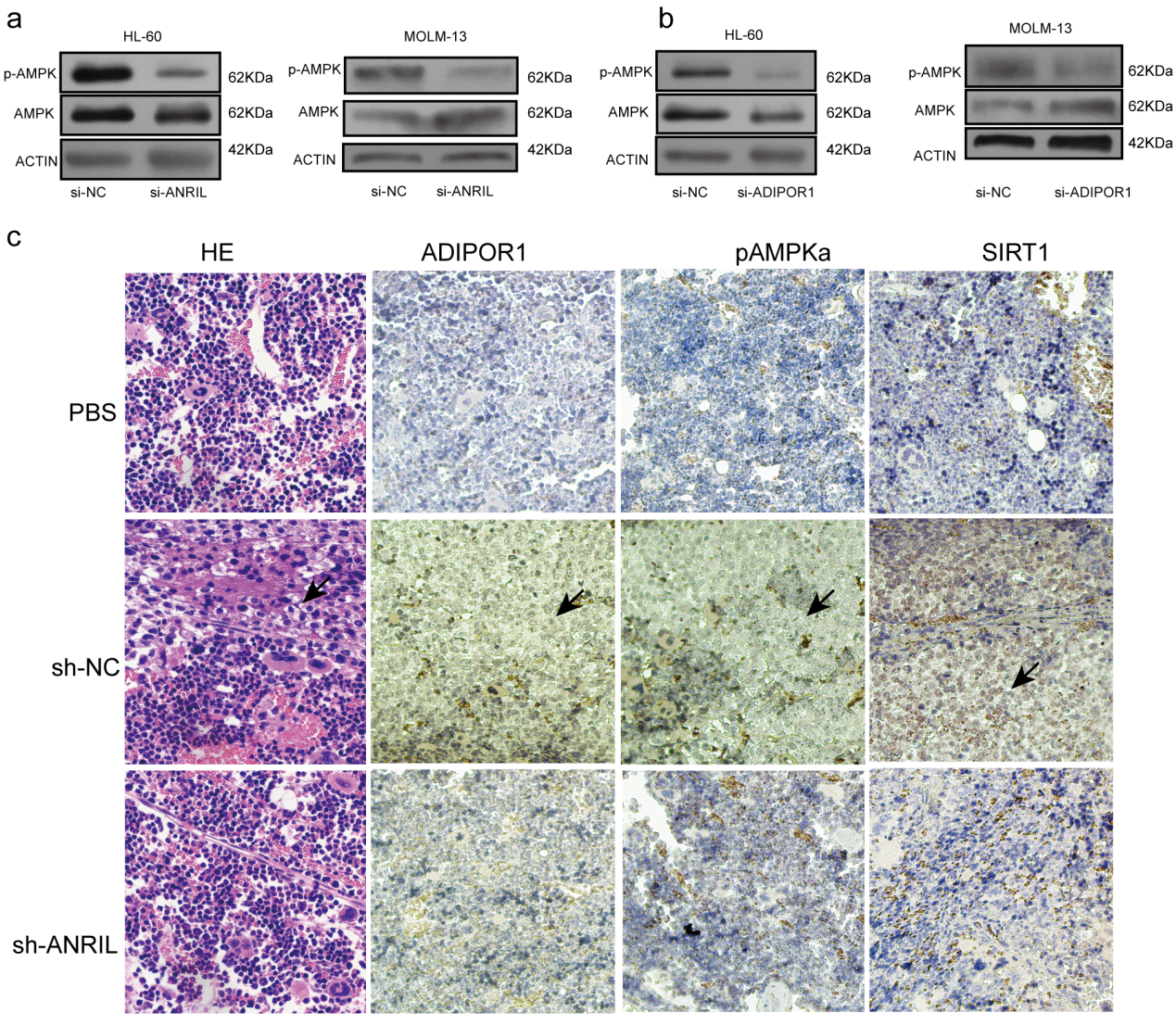
Figure S5 ANRIL regulates the AdiopR1/AMPK/SIRT1 signaling pathway.** The western blot for the total AMPK and pAMPK under knockdown of ANRIL (**a**) and ADIPOR1 (**b**) in AML cells. **c** hematoxylin and eosin (H&E) staining has also showed the reduced number of AML cells in the BM from the sh-ANRIL-Molm13 mice, and Immunohistochemistry assay of the ADIPOR1, pAMPKa and SIRT1 protein levels in the BM.

**Table S1 Characteristics of test cohort.**

| **Characteristics** | **Median (range)** | **No. (%)** |
| --- | --- | --- |
| **Prednisone response** |  |  |
| Good response |  | 73 (65.8) |
| Poor response |  | 21 (18.9) |
| N/A |  | 17 (15.3) |
| **AML (N=109)** |  |  |
| **Age at diagnosis, y** | 8.3 (0.1-15) |  |
| **Sex** |  |  |
| Male |  | 61 (55.9) |
| Female |  | 48 (44.1) |
| **WBC count, × 10^9^/L** | 36.1 (1.2–529.9) |  |
| **FAB** |  |  |
| M1 |  | 4 (3.7) |
| M2 |  | 15 (13.8) |
| M3 |  | 64 (58.7) |
| M4 |  | 5 (4.7) |
| M5 |  | 12 (11.0) |
| M6 |  | 2(1.8) |
| M7 |  | 1(0.9) |
| N/A |  | 6(5.5) |
| **Risk group** |  |  |
| HR |  | 39(35.8) |
| MR |  | 36(33.0) |
| SR |  | 21(19.3) |
| N/A |  | 13(11.9) |
| **ITP Control (N=14)** |  |  |
